# Atmosphere-dependent synthesis and crystallization behavior of silica nanoparticles derived from rice husk biomass

**DOI:** 10.1039/d6ra00367b

**Published:** 2026-03-27

**Authors:** Fuad Hama Shareef Radha, Mohammad Tahir Kareem, Omed Gh. Abdullah

**Affiliations:** a Department of Chemistry, College of Science, University of Sulaimani Qliasan St 46001 Sulaymaniyah Kurdistan Region Iraq; b Department of Physics, College of Science, University of Sulaimani Qliasan St 46001 Sulaymaniyah Kurdistan Region Iraq omed.abdullah@univsul.edu.iq

## Abstract

This work on sustainable nanomaterials focuses on their green synthesis methods using cheap and renewable precursors. In this study, silica nanoparticles (SiO_2_ NPs) were prepared from agro-waste biomass rice husk through an environmentally safe extraction process. Three controlled ashing conditions were used to determine the effect of the processing atmosphere on the end-product SiO_2_ NPs. Besides offering a reliable route to silica recovery, this process allowed one to control the phase and structure of the final nanostructures. The formation of high-purity SiO_2_ NPs was confirmed through characterization with the assistance of X-ray diffraction (XRD), Fourier transform infrared (FTIR) spectroscopy, field-emission-scanning electron microscopy (FE-SEM), energy-dispersive X-ray (EDX) spectroscopy, transmission electron microscopy (TEM) and zeta potential measurements. The as-synthesized nanoparticles exhibited a predominantly amorphous nature, with particle diameters ranging from 20 to 37 nm, and consisted of aggregated spherical grains. Post-synthesis thermal treatment at 1000 °C effectively induced a clear amorphous-to-crystalline phase change in the silica structure. This reaction caused the emergence of cristobalite-type silica, confirming that the structural stage and consequent morphology can be accurately engineered by the thermal treatment of the green-synthesised NPs. This study extensively highlights the enormous potential of rice husk biomass as a dual-use feedstock for waste valorization and the scalable production of SiO_2_ NPs, which can be further utilized for phase-associated functional applications.

## Abstract 2

This work on sustainable nanomaterials focuses on their green synthesis methods using cheap and renewable precursors.

## Graphical abstract

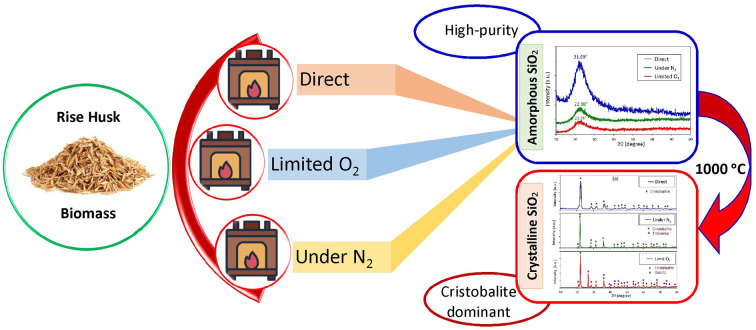

## 1 Introduction

Nanoscience has become a focal point in contemporary materials study, which assists in the design and control of matter at the nanoscale to obtain qualities that cannot be obtained in bulk systems. Advances have been made in energy storage, catalysis, and environmental technologies due to the characteristic properties of nanostructured materials, such as high surface-area-to-volume ratios, high interfacial energy and the quantum confinement effect.^1,2^

Among the various categories of nanomaterials, inorganic nanoparticles are considered one of the most interesting due to their thermal and chemical stability, structural rigidity and adjustable physicochemical properties. The size, shape and structure of particles can be controlled to precisely modulate their properties, which directly affect catalytic efficiency, optical activity and mechanical performance.^3,4^ As a result, recent advances in synthesis methods have been oriented towards the use of methods that are more morphologically precise and environmentally sustainable. These strategies aim at curbing chemical hazards and minimizing energy consumption through the use of renewable raw materials and thus help in the shift towards cleaner and greener pathways for the synthesis of nanomaterials.^5,6^

Various techniques have been developed to fabricate nanoparticles, which are typically classified as top-down or bottom-up procedures.^7^ Despite their ability to achieve controlled synthesis through physical and chemical methods, their economic and environmental sustainability is low because they often demand high energies, expensive equipment, and toxic substances.^8^ Biological techniques involving the use of microorganisms or plant extracts as alternatives are more sustainable in terms of scalability and reproducibility.^9,10^ Green synthesis, in turn, is a promising approach that uses mild reaction conditions and renewable precursors to reduce the use of energy and minimize the amount of chemical waste.^11,12^ Greenly generated nanoparticles may be more stable and active on their surfaces because of the capping or stabilizing agents that are naturally obtained.^8,13^ This green technology is a viable solution for synthesizing silica nanoparticles (SiO_2_ NPs) using natural biomass sources in an eco-friendly manner and does not contradict existing trends in the development of sustainable materials. Silica nanoparticles can be shaped in various shapes, including spheres, rods, and plates. Both types of forms have different surface energies and physicochemical properties.^14^ The ability to tailor these parameters provides a pathway for optimizing the performance of SiO_2_ NPs for particular uses in different industries.^15^ Because of their high thermal stability, SiO_2_ NPs are also used as reinforcing agents in metal and polymer matrix composites, which can improve the mechanical strength and durability of structural materials.^16^

The synthesis of SiO_2_ NPs is highly dependent on the nature of the precursor material used, and as a result, the material selected affects the structure and functional characteristics of that material. Chemical precursors, such as silicon tetrachloride (SiCl_4_), tetraethyl orthosilicate (TEOS) and sodium silicate (Na_2_SiO_3_), have traditionally been the most commonly used precursors, as they are efficient in terms of controlled nanoparticle synthesis but generate hazardous byproducts.^17–21^ At the same time, biosynthesized SiO_2_ NPs through bacteria, fungi, yeast, plant extracts, or algal biomass constitute an eco-friendly, viable solution to traditional chemical processing.^22–24^ The increasing consciousness regarding environmental conservation has led researchers to seek other sources of silica that are more sustainable; accordingly, there have been increased interests in natural renewable sources and silica sources that are industrial wastes, such as wheat husk,^25^ coconut husk ash,^26^ corn cob ash,^27^ rice husk ash,^28,29^ lemon grass,^30^ sugarcane leaves,^31^ St. Augustine grass,^32^ sand,^33,34^ and mineral rocks.^35–37^

These reserves contain amorphous silica sources and can be used to produce nanoparticles that are sustainable to the environment and have a low environmental impact. Although numerous methods of chemical synthesis have been developed, the large-scale manufacture of nano-silica remains an economically problematic issue because of the high cost of materials and energy-consuming steps of the production process. This has prompted scientists to explore less costly and more readily available natural materials as sustainable alternative sources of silica. Silica nanoparticles are widely used across diverse fields due to their highly adaptable surface chemistry, structural stability, and favorable biocompatibility. Within the clinical and diagnostic sectors, researchers have prioritized silica-based nanomaterials for applications in drug delivery, diagnostic imaging, and theranostic platforms, where the pore structure and surface functionality critically influence performance.^38–40^ Consequently, mastering the precision synthesis and structural tailoring of SiO_2_ NPs remains a fundamental challenge in optimizing their performance.

The present study develops a cost efficient yet eco-friendly method to prepare SiO_2_ NPs through a green precipitation process by utilizing locally available rice husk biomass as a renewable silica source. Three methodologies for controlled ashing were employed to determine the role of processing conditions in the size and shape of NPs. Furthermore, the amorphous-crystalline transition was studied in detail. The structural and morphological characterizations were carried out in a comprehensive manner to explain the relationship between the conditions of processing and material properties. The results reveal that rice husk has the potential to be an effective and sustainable precursor in the production of large-scale and phase-controlled synthesis of high-purity SiO_2_ NPs.

## 2 Experimental parts

### 2.1 Materials

All the chemical reagents such as sodium hydroxide (NaOH), hydrochloric acid (HCl), and sulfuric acid (H_2_SO_4_) were of analytical grade and purchased from Merck (Germany). The experiments were conducted using double-distilled water. The silica precursor, rice husk (RH), was gathered in an area rice mill (Chwar Bakh rice mill in Sulaymaniyah City).

### 2.2 Procedure

Silica nanoparticles (SiO_2_ NPs) were prepared from agricultural waste using rice husk waste *via* silica xerogel production. The RH collected at a milling unit was washed twice with running tap water and once more twice with distilled water to clean any dust, flour or contaminants. The dried husk was dried in an oven at 100 ± 5 °C and later placed in airtight containers pending other phases of this research. Three different ashing procedures were employed to convert RH into rice husk ash (RHA), with each process aimed at controlling the oxidizing environment during combustion to a significant degree. In the former method, the material was placed in an open ceramic crucible, burned under normal atmospheric conditions, and left to be completely exposed to oxygen (direct method). The second technique used a partially covered crucible to restrict the oxygen supply, forming a semi-controlled environment that controlled the rate of oxidation (limited O_2_). RH in the third route went through a closed furnace chamber, which was constantly purged with high-purity nitrogen gas (99–99.9%) to create an inert atmosphere (entirely oxygen-free atmosphere) that favored the pyrolytic breakdown of the compound over complete combustion (under N_2_). In all the procedures, all the samples received identical thermal histories and were heated in a muffle furnace at ambient temperatures of 700 °C at a constant heating rate of 10 °C min^−1^ and then 700 °C with a holding time of 4 h to ensure full reaction of the RH to white RHA.

The white ash was first milled into fine powder and then sieved using a sieve of 600 µm. Subsequently, 20 g of rice husk ash (RHA) was placed in a conical flask and combined with 200 mL of 2 M NaOH. The suspension was refluxed at 80–90 °C for 2 h using a heating mantle with continuous stirring to extract silica from the RHA in the form of sodium silicate. Stoichiometric proportions of RHA, NaOH, and HCl were used to facilitate silica precipitation. The extraction of silica was followed by two consecutive steps: (i) dissolution in alkali and (ii) precipitation by acid. The first step involved the dissolution of the RHA in strong alkali (NaOH) to dissolve the amorphous silica, which formed an aqueous solution of sodium silicate (Na_2_SiO_3_).(RHA) SiO_2_ + 2NaOH (aq.) → Na_2_SiO_3_ (aq.) + H_2_O (aq.)

After separation from the solid residue, the alkaline filtrate containing sodium silicate (Na_2_SiO_3_) was slowly neutralized with dilute 0.05 N HCl to a final pH of 7. This gradual pH adjustment triggered the acid-driven precipitation step, leading to the formation of a colloidal silica hydrogel.Na_2_SiO_3_ (aq.) + 2HCl (aq.) → SiO_2_ (gel) + 2NaCl (aq.) + H_2_O (aq.)

**Fig. 1:**
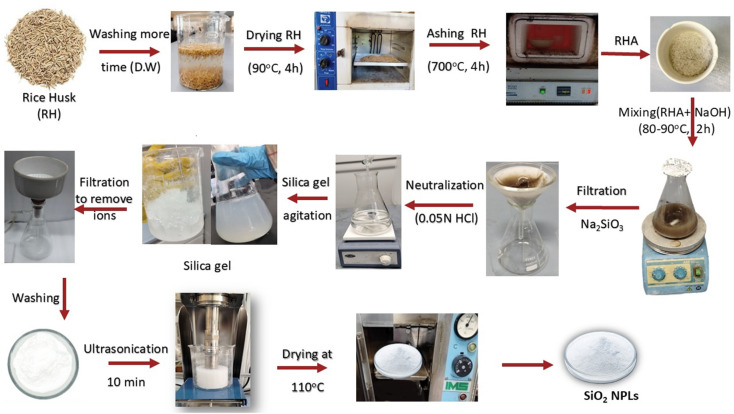
Schematic of the thermo-chemical and post-processing routes in the production of silica nanoparticles from RH.

The neutralization step is very important for enhancing the formation and subsequent polycondensation of the reactive groups of silanol (Si–OH). This sol–gel reaction leads to the development of a three-dimensional Si–O–Si network that characterizes the silica gel structure.^41^ The hydrogel slurry formed at the end of the precipitation using an acid underwent further processing to produce the final silica product. The slurry was allowed to age for 30 h to ensure complete gelation and network development, followed by filtration. The collected gel was repeatedly washed using distilled water to remove soluble impurities, particularly NaCl generated during neutralization. The silica gel was then purified and ultrasonicated to disrupt residual agglomerates and enhance the dispersion of the nanoparticles to yield particles in the size range of 20–100 nm. Subsequently, the silica was dried in an oven at a temperature of 110 °C for 12–24 h to dry up the remaining moisture. Fig. 1 depicts a schematic representation of the production process of the SiO_2_ NPs. A portion of the dried silica was sintered at 1000 °C in a muffle furnace to promote densification and crystallization for structural evaluation. Finally, the process efficiency was assessed by calculating the percentage recovery of RHA and the extracted silica using the following equations:1
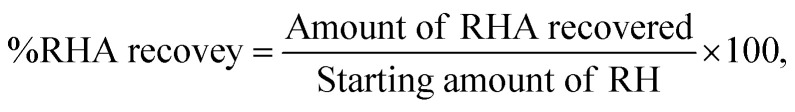
2
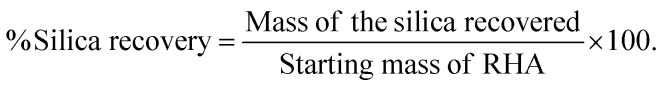


The calculated data were organized, as presented in Table 1. These results highlight the importance of the oxidation environment on the mass yield of rice husk ash (RHA) and silica recovery efficiency. The highest RHA yield (39.1%) was obtained with ashing under N_2_ atmosphere, and it can be attributed to restricted oxygen availability that promotes pyrolytic degradation of organic matter and minimizes mass loss. However, silica recovery from this ash was the lowest (40.2%), indicating that the resulting ash structure exhibits reduced chemical reactivity. Conversely, direct ashing under ambient oxygen conditions produced the highest silica recovery efficiency (98.1%), with moderate RHA recovery (28.7%). This suggests that complete oxidation enhances the accessibility and reactivity of silica, thereby improving its subsequent chemical extraction. Therefore, maximizing the overall silica yield requires prioritizing extractability (high silica recovery) over the raw mass recovered from the initial ashing step. The reduced silica recovery observed under limited O_2_ and N_2_ atmospheres can be attributed to incomplete oxidation of carbonaceous species, resulting in carbon-rich residues that limit silica extractability. In contrast, the direct method maximizes silica yield per unit precursor and avoids the need for controlled atmospheres, thereby reducing operational complexity, energy demand, and associated emissions. Thus, direct calcination represents a more resource-efficient and comparatively lower process-burden pathway for silica extraction than the limited O_2_ and under N_2_ atmosphere.

**Table 1:** Recovery percentages of RHA and extracted silica

Ashing methods	% RHA recovery	% Silica recovery
Direct	28.7	98.1
Limited oxygen	25.8	58.6
Under nitrogen	39.1	40.2

### 2.3 Characterization techniques

Fourier-transform infrared (FTIR) spectroscopy was applied to monitor the chemical bonds present in the SiO_2_ NPs and to confirm the removal of organic residues. The analysis was conducted using a PerkinElmer spectrometer (Model 2000) and Thermo Scientific Nicolet (Model: iS10), over the wavenumber range of 400–4000 cm^−1^. Prior to measurement, the specimens were mixed with spectroscopic-grade potassium bromide (KBr) and pressed into transparent pellets across the relevant vibrational range.

X-ray diffraction (XRD) measurements were conducted using a Philips-PW1730 diffractometer equipped with a Cu anode (*λ* = 0.154 nm) operated at a maximum power of 2.2 kW and a voltage of 60 kV. The system utilized a long fine-focus ceramic tube, and diffraction patterns were recorded within a 2*θ* range of 10°–80° with a constant scan rate of 5° min^−1^. These data were used to determine the crystalline phase of the prepared SiO_2_ NPs and to estimate their average crystallite size.

A field emission-scanning electron microscope (FE-SEM) was used to assess the surface morphology of the prepared SiO_2_ NPs; microstructural imaging was conducted using a TESCAN MIRA3 produced in Czechia. Prior to FE-SEM imaging, the samples were sputter-coated with a thin gold layer to enhance electrical conductivity and minimize charging effects, thereby ensuring stable, high-contrast micrographs. Elemental analysis was conducted using energy-dispersive X-ray spectroscopy (EDX) integrated within the FE-SEM system. High-resolution FE-SEM images were first acquired to identify regions of interest for elemental mapping. EDX software was subsequently employed to generate color-coded elemental maps illustrating the surface distribution of the constituent elements. Transmission electron microscopy (TEM) analysis was performed using a PHILIPS CM120 microscope to investigate the morphology and particle size distribution of the SiO_2_ NPs. For TEM analysis, a small amount of the SiO_2_ powder was ultrasonically dispersed in alcohol, and a drop of the suspension was deposited onto a carbon-coated copper grid and allowed to dry before imaging.

The zeta potential of synthases SiO_2_ NPs using different ashing methods was measured by dispersing the nanoparticles in deionized water. Zeta potential measurements were performed using a Malvern Zetasizer Nano instrument at 25 °C.

The specific surface area (SSA) of the synthesized SiO_2_ NPs was estimated using the methylene blue (MB) adsorption method. In this method, a known mass of SiO_2_ (0.1 g) was dispersed in an aqueous solution of MB and stirred for 30–60 min until the adsorption equilibrium was reached. The decrease in dye concentration was determined by UV-Vis spectrophotometry at 664 nm. The amount of adsorbed MB was calculated. Assuming monolayer adsorption of MB molecules on the particle surface, the SSA was estimated from the quantity of MB adsorbed and the known molecular cross-sectional area. This method provides a practical indirect estimation of SSA.

## 3 Results and discussion

### 3.1 FTIR analysis

**Fig. 2:**
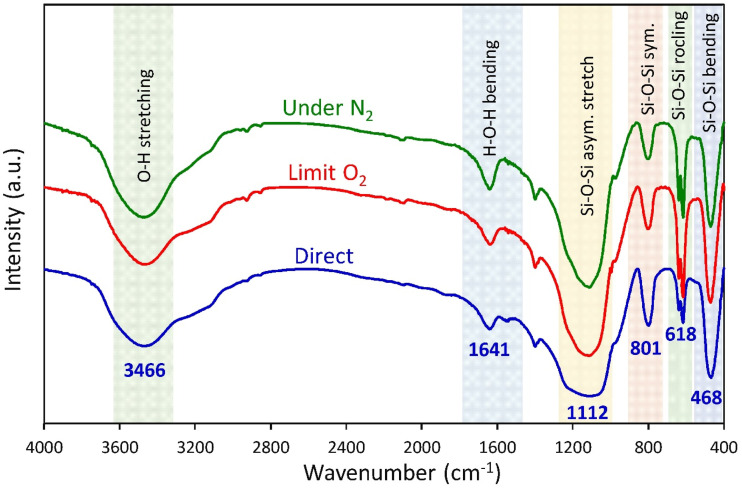
FTIR spectra of the SiO_2_ NP powder synthesized from RH using different ashing methods.

The FTIR spectra of the SiO_2_ NP powder produced from rice husk using different ashing methods are presented in Fig. 2. The three FTIR spectra of silica samples generally exhibit a broad absorption band around 3466 cm^−1^, which corresponds to the O–H stretching vibration of silanol (Si–OH) groups and physically adsorbed water molecules on the silica surface.^27,31^ A characteristic peak near 1641 cm^−1^ is consistently observed, which is attributed to the bending vibration of water molecules either trapped within the silica matrix or associated with Si–OH groups.^42,43^ These features confirm the presence of hydroxyl functionalities and adsorbed water as inherent characteristics of silica materials.

A vigorous peak at 1112 cm^−1^ was linked to the stretching asymmetric vibration of Si–O–Si; the stretching symmetric vibration of Si–O–Si was related to the band of 801 cm^−1^, and the band of 468 cm^−1^ was attributed to the bending vibration of Si–O in siloxane networks.^42^

The enhanced Si–O–Si bending vibration band observed in the limited O_2_ sample can be attributed to modified combustion kinetics during the ashing process. Reduced oxygen availability moderates oxidation, enabling gradual silanol (Si–OH) condensation and promoting the development of a more interconnected siloxane (Si–O–Si) network.^44^ In contrast, fully oxidative conditions may induce rapid combustion and localized thermal effects, which can alter bond distribution within the silica framework. Compared with N_2_ treatment, limited O_2_ provides sufficient oxidative conditions to facilitate silanol condensation while limiting excessive structural damage. Consequently, the resulting higher degree of siloxane crosslinking is reflected in the increased intensity of the Si–O–Si bending vibration.

The band observed near 618 cm^−1^ is commonly assigned to the rocking lattice vibration modes of SiO_2_ cristobalite, resulting from the Si–O–Si framework's rocking vibrations in the ordered tetrahedral network.^45^ The presence of this band in all three samples is therefore considered a spectroscopic indicator of crystalline silica with domains of the cristobalite phase after sintering at high temperatures. The chemical purity of the synthesized silica was also supported by the absence of impurity bands in the range of 1700–2750 cm^−1^. Furthermore, the small band at 975 cm^−1^ in all silica samples, which was assigned to the silanol groups, represented a slight deviation from the commercial silica spectra. The synthesis of silanol and siloxane groups can be associated with the process of hydrolysis and condensation of the acid-neutralized solubilization of sodium silicate. In general, the FTIR spectra support the similarity between the structures of all three derived silicas using different ashing methods. These spectral features confirmed the purity of the synthesized silica using different ashing methods. Table 2 summarizes the important FTIR vibrational modes of as-synthesized SiO_2_ NPs.

**Table 2:** FTIR spectra bands for the synthesized SiO_2_ NPs using the direct ashing method

Wavenumber (cm^−1^)	Vibration type	Assignment	Structural meaning
3466	O–H stretching	Si–OH/adsorbed H_2_O	Surface hydroxyl group
1641	H–O–H bending	H_2_O molecule	Adsorbed water
1112	Si–O–Si asym. stretch	Siloxane bond	SiO_2_ network
801	Si–O–Si sym. stretch	Siloxane	Tetrahedral structure
618	Si–O–Si rocking	Siloxane	Cristobalite SiO_2_ framework
468	Si–O–Si bending	Siloxane	Amorphous SiO_2_ framework

### 3.2 XRD analysis

**Fig. 3:**
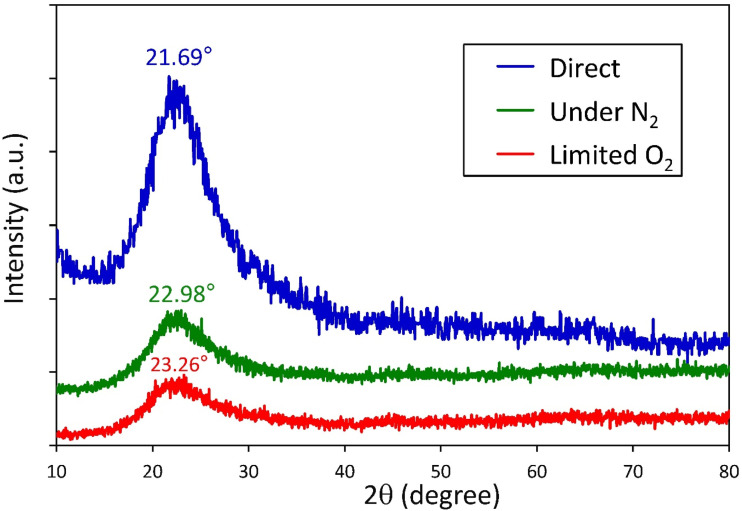
XRD patterns of the SiO_2_ NP powder synthesized from RH using different ashing methods.

The structural features of the synthesized SiO_2_ NPs from RH through different approaches, including direct ashing, limited O_2_ and under N_2_ atmosphere, were examined using XRD, as depicted in Fig. 3. The XRD patterns of all samples exhibited a single broad diffraction halo, characteristic of amorphous silica dried at 110 °C, confirming the absence of sharp diffraction peaks associated with crystalline phases or impurities. The position of this broad peak reflects the most probable inter-atomic correlation distance within the amorphous structure. The shift of this amorphous maximum from 21.69° to 22.98° and 23.26° for synthesized SiO_2_ NPs *via* direct, under N_2_, and limited O_2_ atmosphere, respectively, indicates a systematic reduction in mean inter-atomic spacing, which is consistent with denser local packing. The concurrent intensity loss and peak broadening suggest reduced short-range structural coherence and a higher level of structural disorder.^43,46^

**Fig. 4:**
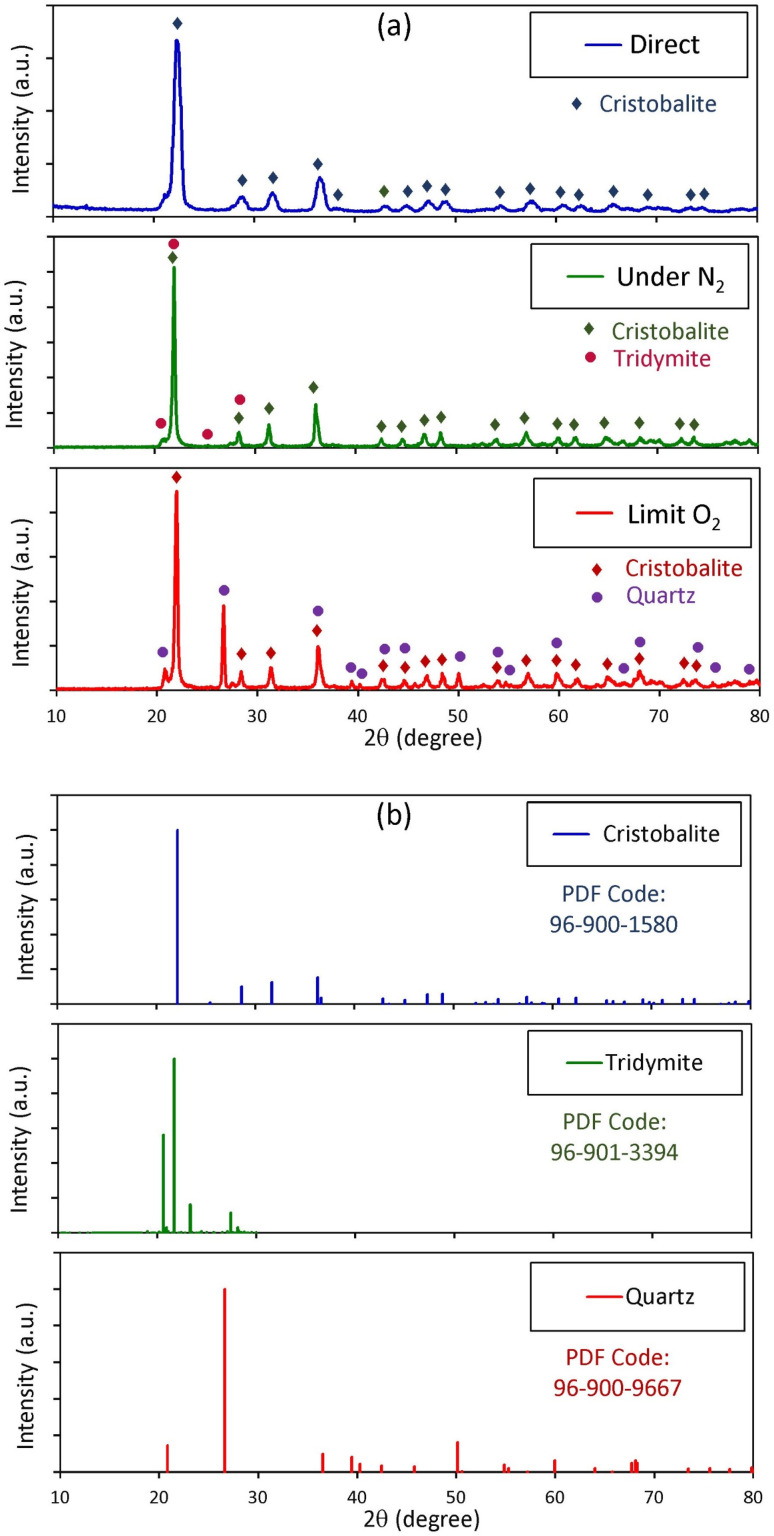
XRD pattern of the (a) SiO_2_ NP powder synthesized from RH using different ashing methods after sintering at 1000 °C and (b) standard reference pattern of the cristobalite, tridymite, and quartz phases.

The XRD patterns of SiO_2_ NP powder after sintering at 1000 °C are presented in Fig. 4, revealing a clear transition from the initially amorphous SiO_2_ NP structure to a crystalline phase. Sintering of SiO_2_ NPs powder prepared by direct ashing induces the emergence of multiple sharp diffraction peaks at 2*θ* = 22.1°, 28.5°, 31.6°, 36.2°, 36.6°, 42.8°, 45.1°, 47.3°, 48.8°, 54.5°, 57.3°, 60.6°, 62.3°, 65.4°, 69.1°, 73.1°, and 74.3°, confirming the development of a long-range order. These diffractions are attributed to crystalline phase planes (011), (111), (012), (020), (112), (121), (022), (113), (122), (023), (031), (131), (032), (132), (124), (133) and (232) of a crystalline tetragonal SiO_2_ cristobalite phase, with cell parameters *a* = *b* = 4.95 Å and *c* = 6.876 Å, in agreement with the reference pattern PDF Code 96-900-1580.^9,47^ Therefore, the XRD results show that the as-extracted amorphous silica is converted into well-crystallized silica through the process of sintering for all SiO_2_ NP powders synthesized from RH using different ashing methods. At high temperatures (1000 °C), the thermal energy is sufficient to promote bond rearrangement, enabling atomic diffusion and reorganization of coordination units and the nucleation and growth of stable crystalline domains. As these nuclei grow, they replace the short-range order of the amorphous SiO_2_ NPs with extended lattice structures, giving rise to increasingly sharp and intense Bragg reflections. The purity of the synthesized SiO_2_ NPs is further affirmed by the absence of extraneous peaks, indicating that the dominant phase is silica. Such amorphous silica and crystalline silica, due to their high surface area and reactivity, are considered highly valuable for a wide range of industrial and environmental applications.^48^

It is clear from Fig. 4 that the sample produced by direct ashing crystallized into a single tetragonal cristobalite phase, indicating that its precursor mixture followed a uniform decomposition and nucleation pathway. The SiO_2_ NPs prepared under N_2_ and limited O_2_ atmosphere after sintering at 1000 °C crystallized into polymorphs of α-cristobalite (PDF Code: 96-900-1580) with tridymite (PDF Code: 96-901-3394) or quartz (PDF Code: 96-900-9667).^49^ It is important to note that the SiO_2_ NP product formed during the direct-ashing process crystallized predominantly into a single cristobalite phase, while the product formed by the limited O_2_ ashing process consisted of two crystalline cristobalite and quartz phases. However, the existence of the cristobalite and tridymite phases was predominant in ashing in a nitrogen atmosphere.^50^ Although the crystallization pathways differ for different ashing methods, the absence of extraneous peaks confirms that all samples remain compositionally pure. However, prepared SiO_2_ NPs under N_2_ and limited O_2_ atmosphere yielded two polymorphic forms rather than impurity-driven phases.

The formation of a single dominant phase in the direct-ashing process and two phases in limited O_2_ and under N_2_ conditions can be attributed to atmosphere-dependent crystallization kinetics. Excess oxygen promotes the rapid and homogeneous combustion of organic residues, resulting in a structurally homogenous amorphous precursor that primarily crystallizes as a single cristobalite phase. However, restricted oxygen availability slows down decomposition, alters defect distribution within the silica network, and increases structural heterogeneity. These factors alter nucleation and growth kinetics during sintering, enabling competitive phase development and the coexistence of secondary crystalline phases. Thus, according to the present results, oxygen control is one of the major parameters in tailoring structural and recovery outcomes.

To assess the influence of the three ashing methods on the structural coherence of the resulting SiO_2_ NPs, the crystallite dimensions were estimated using the Scherrer formula:3
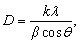
where *D* is the mean crystallite size, *k* is the shape factor constant typically taken as 0.9, *λ* is the X-ray wavelength (*λ* = 1.5406 Å), *β* is the full width at half maximum (FWHM) of the selected diffraction peak, and *θ* is the corresponding Bragg angle. The application of this analysis revealed notable differences among the ashing methods. The SiO_2_ NPs produced using the direct ashing method exhibited an average crystallite size of approximately 11.48 nm, while limited O_2_ and under N_2_ atmosphere ashing yielded markedly larger values of about 25.78 nm and 32.78 nm, respectively. This systematic increase indicates that the thermal and chemical conditions inherent to each ashing route govern the extent of crystallite coarsening during nucleation and growth. The differences in crystallite size among the three ashing routes indicate that the atmosphere of ashing adjustment is an effective lever for tuning the crystallinity of the resulting SiO_2_ NPs.

### 3.3 FE-SEM and EDX analyses

**Fig. 5:**
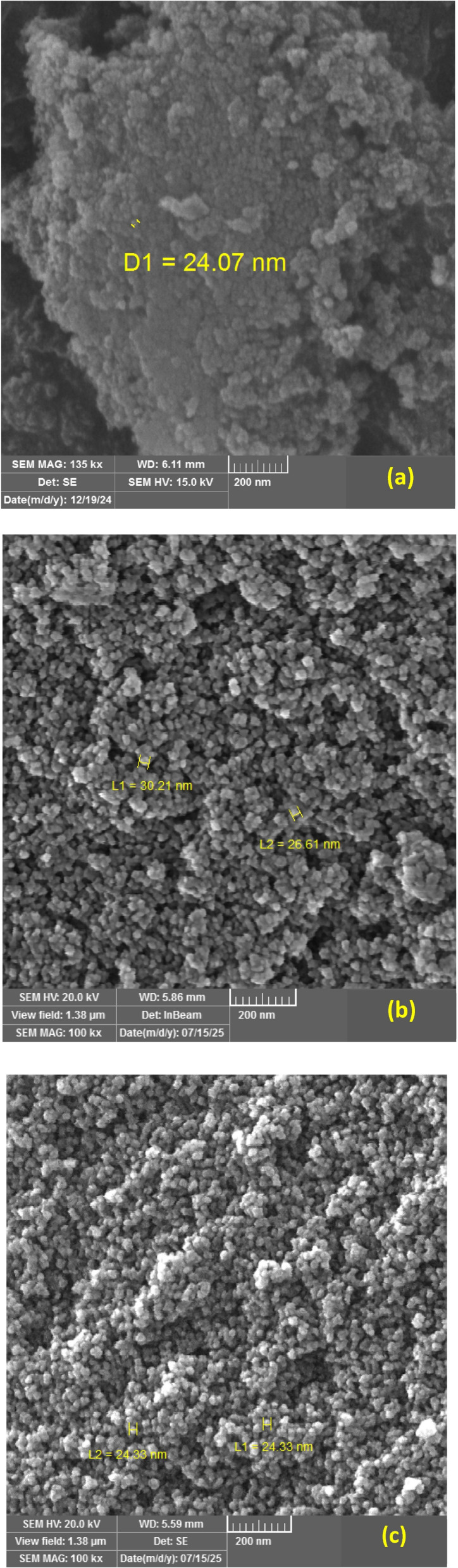
FE-SEM images of the SiO_2_ NP powder synthesized from RH using different ashing methods: (a) direct, (b) limited oxygen, and (c) under nitrogen.

FE-SEM observations confirmed that the SiO_2_ NPs produced through the green synthesis processes using agro-wastes (RH) possessed a predominantly spherical morphology and nanoscale dimensions, with their particle sizes varying systematically according to the specific ashing method used. The FE-SEM micrographs of the SiO_2_ NPs obtained from direct, under N_2_ atmosphere, and limited O_2_ ashing methods are shown in Fig. 5. In all cases, the particles exhibit limited aggregation, forming small clusters while largely retaining their individual morphology. This mild agglomeration is typical of nanoscale ceramics and arises from surface–driven interactions during drying and powder handling.^51^ The partial aggregation observed in the green-synthesized SiO_2_ NPs is anticipated because of their large surface area, and the resulting attractions between nanoparticles promote the formation of small asymmetrical clusters. In agro-waste extracts, the abundance of a large fraction of hydroxyl-bearing organic molecules tends to slow down condensation and hydrolysis reactions, thereby restricting particle growth and reducing the formation of oversized particles. In addition, the diverse phytochemicals present in the extracts act as stabilizing and capping agents, influencing both the stability and aggregation properties of the nanoparticles. These observations underline that the chemical profile of the extract, as well as dispersion conditions during synthesis, play a crucial role in determining nanoparticle size, morphology, and colloidal behavior.^52^

**Fig. 6:**
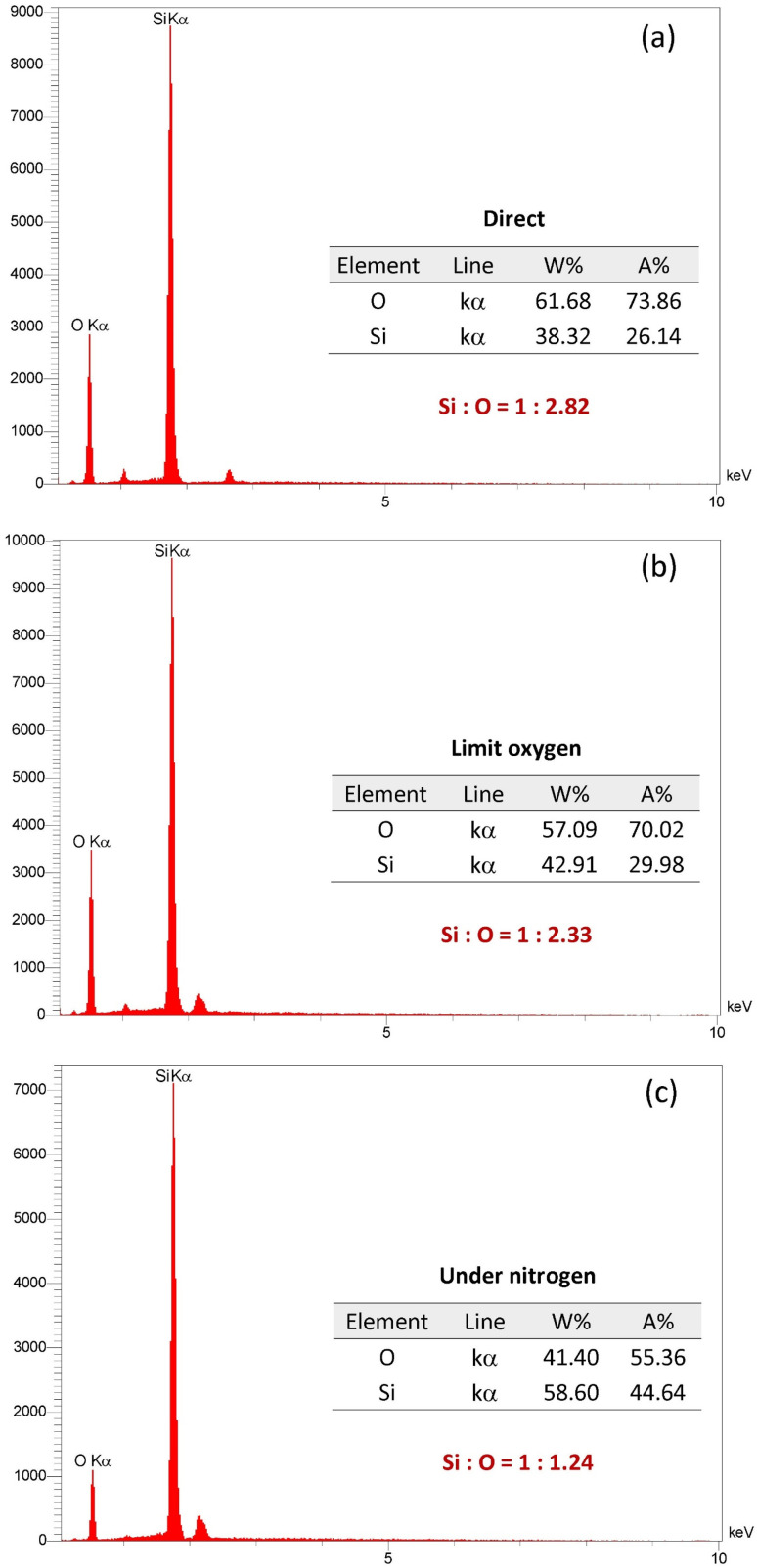
Chemical composition of SiO_2_ NPs extracted from RH using different ashing methods: (a) direct, (b) limited oxygen, and (c) under nitrogen.

The chemical composition of SiO_2_ NP powder extracted from RH using different ashing methods was determined using EDX spectroscopy, as shown in Fig. 6. EDX results confirmed that the as-prepared SiO_2_ NPs using the three ashing methods, direct, limited O_2_, and under N_2_ atmosphere, exhibit high elemental purity. In all samples, the SiO_2_ signal dominated the spectrum, corresponding to a purity level exceeding 98 wt%. Minor signals associated with trace elements were also detected; these are attributed either to residual precursors or to unavoidable background contributions from the substrate and coating. The elemental composition of the synthesized SiO_2_ NPs using the direct method is summarized in Table 3. Although the samples exhibit high purity SiO_2_, small amounts of residual impurities were still detectable after the extraction. The close similarity of the EDX profiles across the three ashing approaches indicates that the ashing atmosphere does not markedly influence the elemental composition and that all methods yield SiO_2_ NPs with comparable chemical cleanliness. EDS analysis confirmed that silica constituted the primary phase across all samples. The calculated atomic ratios of Si : O for the three prepared, direct, limited O_2_, and under N_2_ are 1 : 2.82, 1 : 2.33, and 1 : 1.24, respectively. Although the first two methods yielded oxygen levels slightly exceeding the theoretical stoichiometric ratio of 1 : 2, the third method resulted in a sub-stoichiometric oxygen content. The elevated oxygen values in the direct and limited O_2_ samples are likely due to the common overestimation of oxygen in EDS measurements, as well as the presence of surface hydroxyl groups in silica materials, which is consistent with our earlier FTIR analysis. Conversely, the oxygen deficiency observed in the under N_2_ sample can be attributed to the inert synthesis atmosphere, which significantly limited the O_2_ availability during the ashing method.

**Table 3:** Elemental composition of SiO_2_ NPs extracted from RH using the direct method

Elements	O	Si	Na	Mg	Al	S	Cl	K	Ca	Fe
wt%	63.33	34.7	0.21	0.0	0.19	0.14	0.04	0.09	0.21	0.11

### 3.4 TEM analysis

**Fig. 7:**
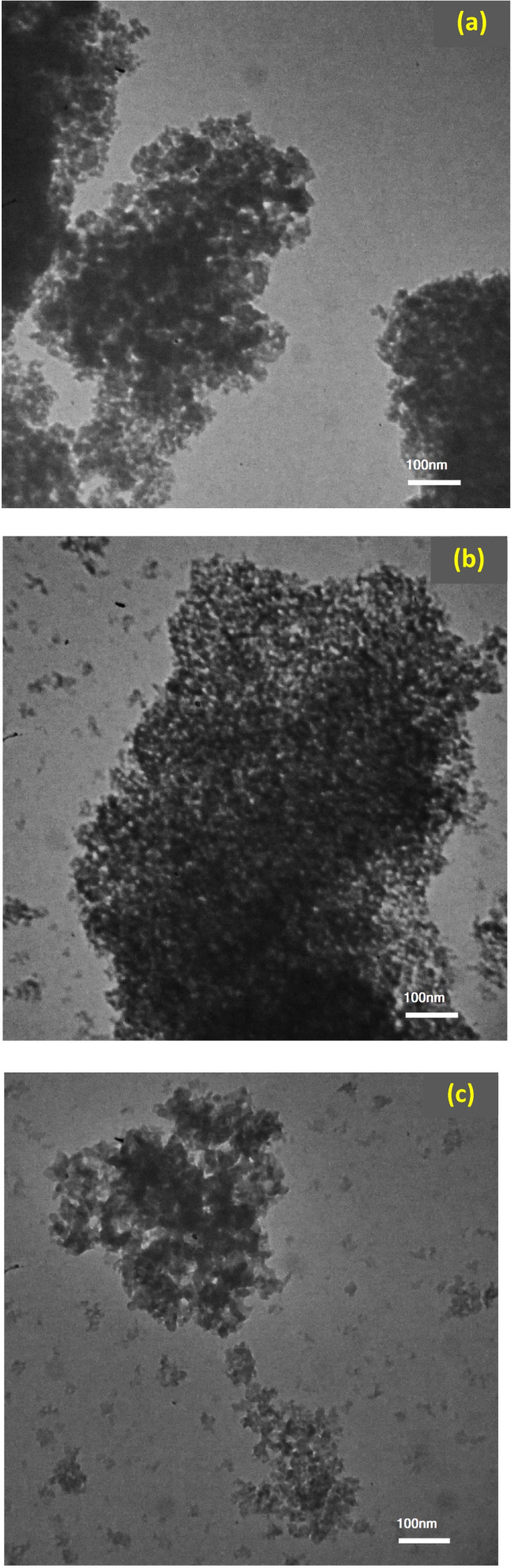
TEM images of SiO_2_ NPs extracted from RH using different ashing methods: (a) direct, (b) limited oxygen, and (c) under nitrogen.

**Fig. 8:**
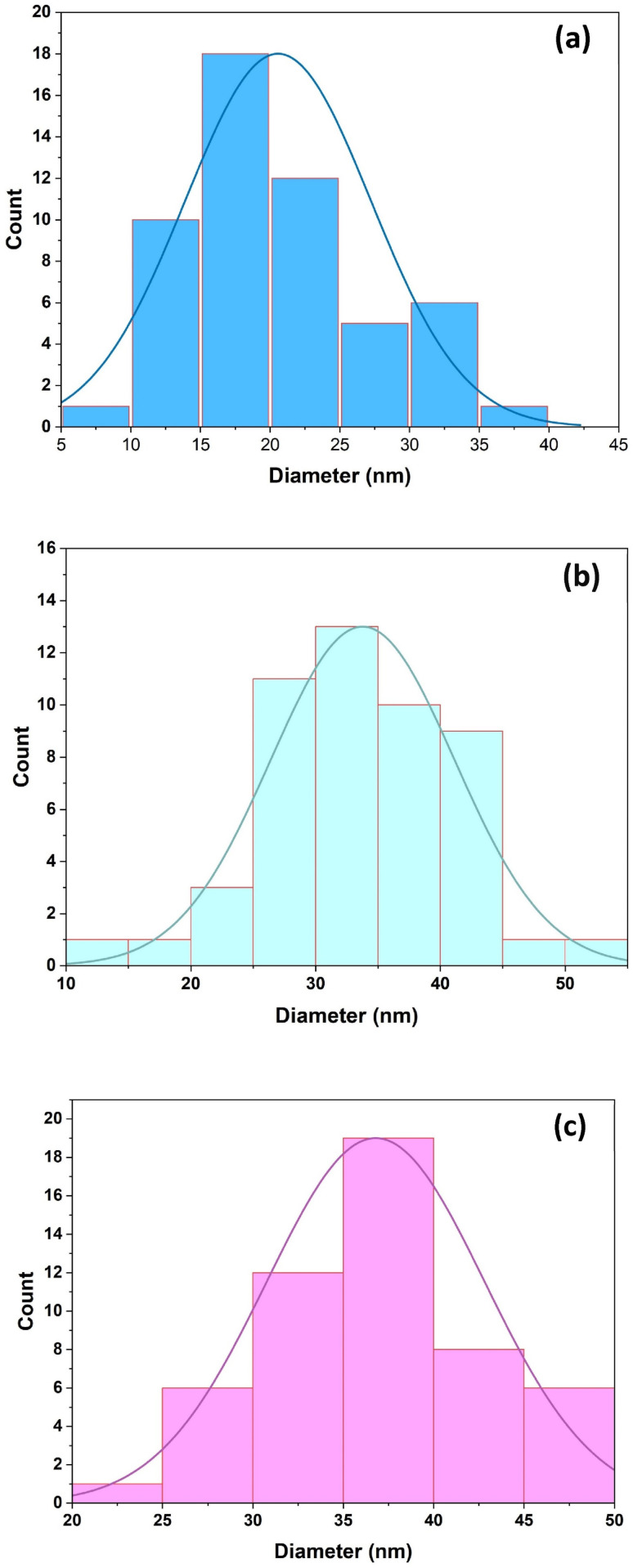
Particle size distribution of SiO_2_ NPs extracted from RH using different ashing methods: (a) direct, (b) limited oxygen, and (c) under nitrogen.

TEM was employed to visualize the morphology and size of the synthesized SiO_2_ NPs extracted from RH through different ashing routes, while particle-size distributions were obtained by determining representative particles in ImageJ. The TEM micrographs of SiO_2_ NPs extracted from RH through different ashing routes are presented in Fig. 7. The particle-size distributions derived from the TEM image analysis are depicted in Fig. 8. The obtained results reveal clear variances in nanoparticle size and dispersion according to the ashing methods. The histograms show a progressive shift toward larger SiO_2_ NP sizes upon shifting from the direct ashing method to the ashing method in an N_2_ atmosphere. The SiO_2_ NPs obtained using the direct ashing method are generally the smallest and exhibit a more uniform appearance with an average diameter close to 20 nm, while the limited O_2_ method yields slightly larger nanoparticles with a broader spread in SiO_2_ NP size with an average diameter close to 34 nm. Ashing under N_2_ atmosphere produces the largest SiO_2_ NPs among the three sets (37 nm), and a greater degree of size variation. The observed particle-size distributions obtained using the direct ashing method are consistent with earlier reports on synthesized SiO_2_ NPs from fly ash^53^ and porous volcanic rock,^54^ exhibiting comparable morphology and size characteristics. It is noteworthy that the TEM-derived particle sizes across the three ashing processes exhibit the same growing trend as the crystallite sizes calculated from the XRD data. This consistency between morphological and structural tests supports the synthesis pathway's impact on the formation of SiO_2_ NPs.

### 3.5 Zeta potential analysis

**Fig. 9:**
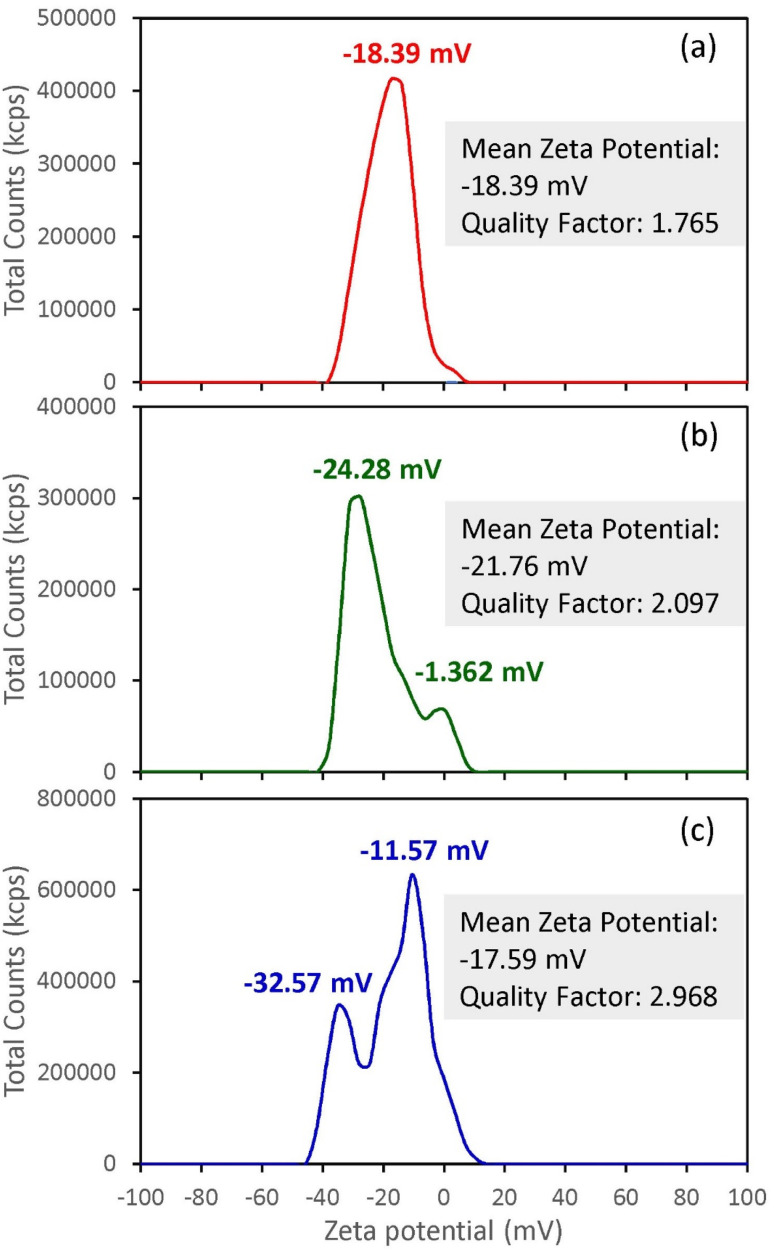
Zeta potential of SiO_2_ NPs extracted from RH using different ashing methods: (a) direct, (b) limited oxygen, and (c) under nitrogen.

Zeta potential was measured to assess the electrostatic stability and aggregation tendency of the synthases SiO_2_ NPs, which directly influence dispersion homogeneity and subsequent processing behaviour.^52,55^Fig. 9 presents the zeta potential measurements of SiO_2_ NPs in deionized water. The zeta potential values for all the synthases SiO_2_ NPs were negative, consistent with previous studies, which have shown that SiO_2_ exhibits a negative surface charge under neutral conditions, indicating colloidal stability in aqueous dispersions.^56,57^

Zeta potential measurements revealed a negative surface charge of −18.39 mV for the direct ashing method SiO_2_ NPs dispersed in water, indicating moderate electrostatic stability due to the deprotonated Si–OH groups, as confirmed previously in the FTIR analysis section. The unimodal distribution confirms a homogeneous surface charge without evidence of secondary aggregation. The slightly less negative zeta potential compared to amorphous silica is caused by the crystalline phase of the prepared SiO_2_ NPs. However, the zeta potential distribution exhibited a bimodal profile with two peaks centered at −24.28 mV and −1.362 mV for ashing with limited O_2_, and −32.57 mV and −11.57 mV for ashing under N_2_, indicating the presence of two electrokinetically distinct nanoparticle populations. The more negative population in the two methods corresponds to well-dispersed SiO_2_ NPs exhibiting good electrostatic stability, while the secondary population at less negative potentials suggests partially aggregated or weakly charged clusters. These results agree well with the TEM results, where the direct ashing method showed the smallest SiO_2_ NPs, while ashing under nitrogen showed a maximum nano size, indicating the aggregation of SiO_2_ NPs in this ashing method.

### 3.6 Specific surface area analysis

The specific surface area (SSA) of as-synthesized SiO_2_ NPs using different ashing methods and after sintering at 1000 °C was estimated using the MB adsorption method, a widely used approach for evaluating the surface area of porous powders and nanostructured materials.^58^ The SSA values for as-synthesized SiO_2_ NPs using different ashing methods are presented in Table 4. The calculated values of SSA were 117.40, 116.69, and 115.48 m^2^ g^−1^ for amorphous silica obtained by direct, limited oxygen, and nitrogen atmosphere ashing methods, respectively, corresponding to MB adsorption values of 1.500 × 10^−5^, 1.491 × 10^−5^, and 1.475 × 10^−5^ mol. These results are in good agreement with other published results for amorphous SiO_2_ NPs prepared using different methods.^59,60^

**Table 4:** Specific surface area (SSA) of the as-prepared SiO_2_ NPs and after sintering at 1000 °C

Ashing methods	SSA of the as-prepared sample (m^2^ g^−1^)	SSA after sintering (m^2^ g^−1^)
Direct	117.40	14.71
Limited oxygen	116.69	13.83
Under nitrogen	115.48	13.46

After sintering at 1000 °C, the SSA values of SiO_2_ powder significantly decreased to 14.71, 13.83, and 13.46 m^2^ g^−1^, with corresponding MB adsorption values of 1.880 × 10^−6^, 1.768 × 10^−6^, and 1.720 × 10^−6^ mol, respectively. The pronounced decrease in the surface area of SiO_2_ NPs after sintering can be explained by transforming the amorphous phase into crystalline phases, usually cristobalite, along with sintering, pores collapse and condensation of surface silanol groups at high temperatures. These observations are consistent with the structural features revealed by the XRD and TEM analyses of the samples.

Thus, crystallization caused densification of the silica framework and a decrease in SSA. The results generally reveal that the surface areas of amorphous SiO_2_ NPs are much larger than those of their crystalline counterparts, which are formed through high-temperature sintering. The use of a sustainable method with a different ashing approach and sintering enabled the successful synthesis of high-purity SiO_2_ NPs from rice husk with a wide range of specific surface areas from 117.40 m^2^ g^−1^ for amorphous to 13.46 m^2^ g^−1^ for crystalline phase, indicating its promising potential for applications in catalysis and polymer-based technologies.

## 4 Conclusion

This study demonstrates that ashing environments of agro-waste biomass offer considerable flexibility for tailoring the characteristics of SiO_2_ NPs of comparable purity but distinct size, crystalline phase and morphological features. FTIR spectra confirmed the absence of residual organic groups in all ashing routes and presented a pure SiO_2_ NPs powder. XRD analysis confirmed that the as-prepared powders maintained an amorphous nature. However, sintering at 1000 °C produced different structural results, producing a single tetragonal cristobalite phase for the sample prepared using the direct ashing method, and mixed phases with dominant cristobalite in a limited oxygen and nitrogen atmosphere during ashing procedures. The impact of the ashing conditions was further demonstrated by microscopy findings. SEM and TEM revealed noticeable differences in particle size and dispersion. The direct ashing method produced the smallest and most uniform particles; however, the limited oxygen and under nitrogen ashing methods showed progressively larger and more heterogeneous dispersions. These trends were corroborated by the particle-size distributions derived from ImageJ analysis. EDX confirmed high elemental purity across all samples, with very few trace signals. The findings indicate that the ashing condition of the agro-waste extraction process has a significant impact on particle size, morphology, specific surface area and phase development, highlighting the necessity of carefully controlling synthesis ashing conditions when customizing high quality SiO_2_ NPs for particular applications.

## Author contributions

Fuad Hama Shareef Radha: data curation, formal analysis, investigation, methodology, validation, visualization, writing – original draft, writing – review & editing. Mohammad Tahir Kareem: conceptualization, supervision, validation, visualization, writing – review & editing. Omed Gh. Abdullah: conceptualization, supervision, validation, visualization, writing – review & editing.

## Conflicts of interest

The authors declare no competing financial or personal interests.

## Data Availability

The data will be made available by the corresponding author upon request.
